# Insights into the Impact of Microbiota in the Treatment of NAFLD/NASH and Its Potential as a Biomarker for Prognosis and Diagnosis

**DOI:** 10.3390/biomedicines9020145

**Published:** 2021-02-03

**Authors:** Julio Plaza-Díaz, Patricio Solis-Urra, Jerónimo Aragón-Vela, Fernando Rodríguez-Rodríguez, Jorge Olivares-Arancibia, Ana I. Álvarez-Mercado

**Affiliations:** 1 Children’s Hospital of Eastern Ontario Research Institute, Ottawa, ON K1H 8L1, Canada; jrplaza@ugr.es; 2 Department of Biochemistry and Molecular Biology II, School of Pharmacy, University of Granada, 18071 Granada, Spain; 3 Instituto de Investigación Biosanitaria IBS.GRANADA, Complejo Hospitalario Universitario de Granada, 18014 Granada, Spain; 4 Faculty of Education and Social Sciences, Universidad Andres Bello, Viña del Mar 2531015, Chile; patricio.solis.u@gmail.com; 5 Department of Nutrition, Exercise, and Sport (NEXS), University of Copenhagen, DK-2200 Copenhagen, Denmark; 6 IRyS Research Group, School of Physical Education, Pontificia Universidad Católica de Valparaíso, Valparaíso 2374631, Chile; fernando.rodriguez@pucv.cl (F.R.-R.); jorge.olivares.ar@gmail.com (J.O.-A.); 7 Grupo AFySE, Investigación en Actividad Física y Salud Escolar, Escuela de Pedagogía en Educación Física, Facultad de Educación, Universidad de las Américas, Santiago 8370035, Chile; 8 Institute of Nutrition and Food Technology “José Mataix”, Center of Biomedical Research, University of Granada, Avda. del Conocimiento s/n. Armilla, 18016 Granada, Spain

**Keywords:** non-alcoholic steatohepatitis, intestinal permeability, microbiota, probiotics, physical exercise, fecal microbiota transplantation

## Abstract

Non-alcoholic fatty liver disease (NAFLD) is an increasing cause of chronic liver illness associated with obesity and metabolic disorders, such as hypertension, dyslipidemia, or type 2 diabetes mellitus. A more severe type of NAFLD, non-alcoholic steatohepatitis (NASH), is considered an ongoing global health threat and dramatically increases the risks of cirrhosis, liver failure, and hepatocellular carcinoma. Several reports have demonstrated that liver steatosis is associated with the elevation of certain clinical and biochemical markers but with low predictive potential. In addition, current imaging methods are inaccurate and inadequate for quantification of liver steatosis and do not distinguish clearly between the microvesicular and the macrovesicular types. On the other hand, an unhealthy status usually presents an altered gut microbiota, associated with the loss of its functions. Indeed, NAFLD pathophysiology has been linked to lower microbial diversity and a weakened intestinal barrier, exposing the host to bacterial components and stimulating pathways of immune defense and inflammation via toll-like receptor signaling. Moreover, this activation of inflammation in hepatocytes induces progression from simple steatosis to NASH. In the present review, we aim to: (a) summarize studies on both human and animals addressed to determine the impact of alterations in gut microbiota in NASH; (b) evaluate the potential role of such alterations as biomarkers for prognosis and diagnosis of this disorder; and (c) discuss the involvement of microbiota in the current treatment for NAFLD/NASH (i.e., bariatric surgery, physical exercise and lifestyle, diet, probiotics and prebiotics, and fecal microbiota transplantation).

## 1. Introduction

Non-alcoholic fatty liver disease (NAFLD), which is characterized by an increase in fat accumulation in the form of micro and macro vacuoles of lipids into hepatocytes (named steatosis), is the most common liver disorder worldwide [1]. Steatosis is classified as mild (5–33%), moderate (34–66%), or severe (more than 66%) [2] depending on the fat number in vacuoles within the cytoplasm of hepatocytes. Additionally, other histopathological features should be taken into account in the presence of steatosis including inflammation, fibrosis, and ballooning degeneration [3]. Indeed, NAFLD comprises a large pathological spectrum ranging from simple steatosis to steatohepatitis with a variable degree of fibrosis and cirrhosis [4]. Importantly, liver steatosis is also a critical part of the evaluation of donors’ livers because it is very frequent and may negatively impact recipient outcomes [3,5].

The worldwide prevalence of NAFLD varies widely from 20% to 30% [6] in western countries and increases with age [7]. This disease represents a major health problem because it is associated with the risk of progression to liver cirrhosis, liver cancer, and even increased cardiovascular and solid neoplasm risk [8]. Metabolic syndrome (MetS) is a cluster of metabolic abnormalities including glucose intolerance/insulin resistance, abdominal obesity, atherogenic dyslipidemia, increased blood pressure, a proinflammatory, and a prothrombotic state [9]. MetS provokes the alteration of the metabolic homeostasis of several organs including the liver [10]. The development of NAFLD is strongly associated with MetS given the fact that approximately 90% of the patients with NAFLD have more than one feature of MetS [9]. Glucose and triglycerides, both considered key components of MetS, are overproduced by the fatty liver [11]. In addition, insulin resistance is implied in failure in the formation and utilization of free fatty acids, which ultimately induces steatosis [12]. The liver is therefore a key player of metabolic abnormalities associated with MetS [11]. Unfortunately, obesity and type 2 diabetes (T2DM) are frequently observed in the general population. Evidence suggests that insulin resistance is the major factor associated with steatosis and potentially steatohepatitis, which may precede the development of T2DM. In fact, subjects suffering from NAFLD typically meet the diagnostic criteria for MetS [13]. However, not all patients with these conditions have NAFLD and not all patients with NAFLD suffer from one of these conditions [14]. In this regard, approximately 10% to 25% of people suffering NAFLD can progress to non-alcoholic steatohepatitis (NASH) [7], a more serious form of NAFLD which notably raises the possibilities of progression to cirrhosis and hepatocellular carcinoma (HCC). Besides, it is estimated that 10–15% of patients with NASH will develop HCC [15]. Bearing in mind the aforementioned, NAFLD in general and particularly NASH are ongoing global problems for public health systems. Nevertheless, the exact reason why some patients only develop steatosis and others progress to develop steatohepatitis and fibrosis is not completely understood [14]. It is probably the result of multiple metabolic abnormalities in the setting of a genetic predisposition, being the initial step of the accumulation of fat in the hepatocytes. When chronic NAFLD appears, lobular inflammation and hepatocellular damage can occur, leading to NASH. Moreover, NASH can be described as a necro-inflammatory complication of persistent hepatic steatosis [4]. In recent years, it has been mentioned and described that NAFLD might result from multiple hits with parallel consequences, such as gut and adipose tissue-derived factors [16]. The “first hit” is characterized by insulin resistance, leading to the accumulation of fat in hepatocytes, augmented lipid peroxidation and absolute free fatty acid uptake in the liver, free fatty acid synthesis from cytosolic substrates, the formation of triglycerides, and decreased apolipoprotein B-100 synthesis [17]. The “second hit” occurs through oxidative stress, which describes the progression to liver fibrosis. Reactive oxygen species can stimulate hepatocellular injury through the inhibition and inactivation of mitochondrial respiratory chain enzymes, membrane sodium channels, and glyceraldehyde-3-phosphate dehydrogenase. Reactive oxygen species promote lipid peroxidation, Fas Ligand induction, and cytokine production, adding fibrosis and hepatocellular injury [17]. Tumor necrosis factor-alpha (TNF-α) is up-regulated in NASH, which stimulates other pro-inflammatory cytokines such as interleukin (IL)-6, transforming growth factor beta (TGF-β), and platelet-derived growth factor, as well as augments insulin resistance [18]. On the other hand, a second and most recent theory is the “Multiple-hit” theory, which suggests that inflammation occurs before steatosis in environmentally and genetically predisposed individuals [19]. The “multiple hit” hypothesis contemplates multiple factors operating in genetically predisposed subjects to provoke NAFLD; such factors comprise insulin resistance, nutritional factors, hormones secreted from the adipose tissue, genetic and epigenetic factors, and gut microbiota [20].

Other alterations in gut microbiota (i.e., overnutrition, genetic factors or inadequate lifestyle patterns) [21] have also been reported to promote the development of NAFLD/NASH by mediating inflammation, insulin resistance, bile acids, and choline metabolism [22]. Increased intestinal permeability may be associated with inflammatory changes in NASH due to the strong link between the two processes [23]. The activation of inflammation due to both dysbiosis and alterations in intestinal permeability via toll-like receptor (TLR) signaling in hepatocytes induces progression from simple steatosis to NASH [24]. Furthermore, endotoxin levels were observed to be higher in NAFLD patients with severe fibrosis than in those with mild fibrosis, revealing that the mechanism of fibrotic progression via the endotoxin in NAFLD may be closely associated with gut permeability. Additionally, in patients with NASH, a higher prevalence of small intestinal bacterial overgrowth [25] has been associated with higher levels of TNF-α [26] related to increased expression of TLR-4 on CD14-positive monocytes and higher plasma IL-8 levels [27]. However, neither the pathophysiology of NAFLD/NASH nor the gut microbiota alterations in these patients have been totally characterized, although modulation of the gut microbiota should be a sustainable strategy to manage NAFLD/NASH and comorbidities. Accordingly, strategies such as weight loss via diet and routine modifications are positive commendations to ameliorate liver damage [28]. On the other hand, physical exercise is an additional tool with potential benefits to microbiota composition, gut barrier integrity, and the metabolic profile, including a reduction in the adiposity profile and improvement of inflammation and immune parameters [28].

Concerning diagnosis, liver biopsies remain the gold standard for histological evaluation despite their limitations, invasiveness, and economic cost [29]. More accurate, cheaper, and non-invasive methods are needed for determining the extent of steatosis and its potential in progressing to a more severe status.

The present review summarizes human and animal studies aimed to determine the impact of the gut microbiota alterations in NASH and their suitability as biomarkers for prognosis and diagnosis. Finally, we discuss the implication of microbiota in the current treatments for NAFLD/NASH.

## 2. Diagnosis and Monitoring of NAFLD/NASH

NASH is the active form of NAFLD, with hepatic necroinflammation and faster fibrosis progression. The increasing number of patients that develop NASH-related end-stage liver disease makes it mandatory to establish NAFLD and NASH biomarkers for prognostication and selection of patients for treatment and monitoring [30]. Therefore, it is of critical importance to take into account histological characteristics such as the degree of steatosis, necroinflammation, and fibrosis to evaluate NAFLD patients [30].

Nowadays, liver biopsies remain the gold standard for histological evaluation, although both invasive and non-invasive methods are currently used to detect and track NAFLD and NASH [30]. Liver biopsies are used by clinicians to measure how much ballooning, inflammation, and scarring have occurred in the liver using different scoring scales and algorithms to classify the stages of NAFLD [31]. Nonetheless, this technique presents limitations such as the potential of sampling errors, interpretation of the histopathology, and unsuitability for continuous monitoring and invasiveness [30]. To note, non-invasive imaging techniques have improved over time and may be used to help determine the progression of steatosis, fibrosis, and liver stiffness [31]. Examples of well-established techniques are conventional ultrasonography (the most commonly used to identify fatty liver), computed tomography, or magnetic resonance imaging as well as newer imaging technologies, such as ultrasound elastography, quantitative ultrasound techniques, magnetic resonance elastography, and magnetic resonance-based fat quantitation techniques [32]. On the other hand, standard liver function tests (e.g., elevated liver-associated enzymes such as aspartate aminotransferase and alanine aminotransferase) have shown low sensitivity and specificity as steatosis markers, graft rejection, liver injury, and a poor correlation with the severity of histopathological findings [33]. Researchers have also addressed developing several types of biomarkers including hormones, pro-inflammatory cytokines and proteins, adipokines, and carrier proteins [34]. For instance, plasma cytokeratin 18 fragment levels are related to hepatocyte apoptosis, representing the most extensively evaluated biomarker of steatohepatitis, although the accuracy is modest. In addition, several gene polymorphisms (such as *PNPLA3* and *TM6SF2*) have been shown to correlate with NAFLD and its severity [30].

In this regard, PNPLA3 is the first locus to be reproducibly and strongly related to susceptibility to steatosis and fibrosis/cirrhosis in liver diseases [35]. Indeed, the PNPLA3 polymorphism, rs738409[G] coding for I148M, induces a decrease in enzymatic activity in the hydrolysis of emulsified triglycerides in hepatocytes [36]. The PNPLA3 protein is a lipase that acts in triglycerides in hepatocytes and in hepatic stellate cells (in retinyl esters). PNPLA3 polymorphism, rs738409[G], provokes the loss of its function and triglyceride accumulation in hepatocytes [35]. By contrast, the present lack of high-throughput studies focused on proteomics or metabolomics to establish novel and reliable diagnostic biomarkers for NAFLD hampers epidemiologic studies [7].

In summary, current biomarkers for detecting, classifying, and tracking different features of NAFLD and NASH (namely, steatosis, necroinflammation, or fibrosis) present limitations due to their lack of accuracy, reproducibility, responsiveness, feasibility or economic cost. This shows the need for more effective, less invasive, and more affordable methods for determining the extent of steatosis and its potential in progressing to a more severe status. Thus, all these challenges have increased the interest in developing novel methods for the diagnosis (biomarkers, gut microbiota, among others) and prediction of the different stages of NAFLD/NASH.

## 3. Microbiota and Non-Alcoholic Steatohepatitis

The microbiome involves all of the genetic information inside the microbiota defined as “the full collection of microbes, i.e., bacteria, fungi, virus, etc., that naturally exists within a particular biological niche such as an organism, soil or a body of water, among others” [28,37]. Indeed, the word “microbiome” is also referred to as the metagenome of the microbiota [38]. The mutual communication between the microbiota and the liver is performed through the portal vein, which transports gut-derived products to the liver, and the feedback of bile and antibodies from the liver to the intestine [28,37]. 

The mucus barrier composition is determined by the microbiota as indicated through studies on germ-free mice that when colonized with the microbiota acquire similar mucus from donors [39]. This probably is due to the capability of goblet cells to sense the presence of bacterial products and to produce Muc2 after triggering the NLRP (Nucleotide-binding oligomerization domain, leucine-rich repeat and pyrin-domain containing proteins)-6-inflammasome pathway [40]. Besides, studies performed in animals have shown alterations in TLR signaling related to the leaky gut syndrome by the action of bacterial lipopolysaccharide (LPS). In humans, modifications in the profile of the gut microbiota associated with alterations in intestinal permeability have also been related to liver disease [28]. In consequence, metabolites produced by the microbiota, the immune system, and the liver show a strategic function in the pathogenesis of alcoholic liver disease and NAFLD/NASH [37]. Gut barrier impairment (which induces intestinal permeability) is the dynamic variable that enables portal influx of pathogen-associated molecular patterns (PAMPs), e.g., LPS, and microbiome-derived metabolites to the liver, activating a pro-inflammatory cascade that exacerbates hepatic inflammation [41].

Metagenomic analyses reveal that NASH and cirrhosis are related to changes in the gut microbiota composition [42,43]. In particular, it has been shown that *Eubacterium rectale* and *Bacteroides vulgates* enhancement is associated with NAFLD, probably through damaging metabolic intermediaries in the altered microbiome of the host [44]. 

Patients with NASH showed augmented intestinal permeability and decreased intestinal bacterial overgrowth that are correlated with the severity of steatosis [45]. Likewise, mice deficient in junctional adhesion molecule A (Jam-A), an essential constituent of tight junctions that regulate the paracellular route of solutes avoiding molecules such as LPS cross the epithelium [46], are also more susceptible to NASH development [47]. Some authors have reported that just below the epithelium there is another cellular barrier-dominated gut vascular barrier that senses the entry to the liver and portal circulation [48,49]. Enteric pathogens such as *Salmonella typhimurium* have proven tactics to evade this gut vascular barrier via delaying the WNT/b-catenin signaling pathway [50]. In this regard, preventing the gut vascular barrier disruption with obeticholic acid treatment might prevent the development of NASH [50]. In addition, a recent study in a heterodimeric integrin receptor has shown that by blocking this integrin receptor known as α_4_β_7_, the recruitment of CD4^+^ T cells to the intestine and liver not only reduces hepatic inflammation and fibrosis but also protects the metabolic imbalances related to NASH [51].

Under healthy conditions, the gut microbiota composition is mostly stable, showing dissimilarities mainly at the species level. However, studies in humans revealed that the microbiota differs in patients with NASH. Accordingly, *Enterobacteria* and *Proteobacteria* present increased abundance relative, whereas anti-inflammatory bacterial strains such as *Faecalibacterium prausnitzii* are diminished [52]. In addition, important variables are related to the gut microbiota in the characterization of NASH, such as the serum bile acid profile and the hepatic gene expression pattern that support an increased bile acid production in NASH patients [53].

A better understanding of the patient microbiota interactions and the response to different actions/managements will be essential for the enhancement of NASH therapies and the elaboration of novel approaches targeting the alterations in microbiota associated with NASH.

## 4. Research Studies on Gut Microbiota and Non-Alcoholic Steatohepatitis

The global burden of NASH is high, affecting one in four adults and presenting a substantial geographic variation in prevalence [54]. Liver-detailed mortality of NASH patients was estimated to be 15.44/1000 person-years. Moreover, the impact of NASH as a cause of liver-related mortality may increase several-fold as the age at onset of disease decreases not just to childhood but to the in-utero state [55]. Indeed, this may potentially increase the duration and progression of liver disease. Another important factor is genetic susceptibility which means and unquestionable causal issue, e.g., despite the much lower daily caloric intake in Central and South America than in North America and western Europe, the prevalence of NASH is higher in these regions probably due to the association of this population with an increased prevalence of the rs738409 G allele of the *PNPLA3* gene [56].

### 4.1. Animal Studies

Animal studies have mostly been performed in rodents, revealing a potential causal role of gut microbiota in NAFLD. Several studies performed in animal models evaluating the implication of several genes in microbiota alteration and NASH pathogenesis have been recently reported. For instance, hepatic MyD88 regulates the production of bioactive lipid compounds that are implicated in glucose, inflammation, and lipid metabolism. The deletion of MyD88 in mice fed with a normal diet provoked changes in the gene expression, plasma, and liver metabolome. Moreover, gut microbes similar to those reported have been observed in diet-induced obesity and diabetic subjects [57].

On the other hand, it is well known that Sirtuin 3 (SIRT3) shows a defensive function against NAFLD by refining hepatic mitochondrial dysfunction [58,59]. SIRT3 knockout mice fed with a chow diet or high-fat diet were used to evaluate the relationship between gut microbiota and SIRT3 in NAFLD development. Results from this study showed that SIRT3 deletion accelerated gut microbial dysbiosis after a high-fat diet with augmented levels of *Desulfovibrio* and *Oscillibacter* and reduced *Alloprevotella* abundance. In addition, these mice had augmented LPS levels and dysfunction of cannabinoid receptor 1 and 2 expressions in both the colon and the liver, which were significantly linked to the variations observed in gut microbiota [58].

The disruption of the gene F11r encoding Jam-A has showed deficiencies in intestinal epithelial permeability in mice fed with a normal diet or a diet high in saturated fat, fructose, and cholesterol. Mice with F11r gene knockout developed NASH features and increased levels of inflammatory microbial taxa related to Firmicutes and *Proteobacteria* compared with wild type mice [47].

To note, contemporary studies have proposed a key function for the inflammasome/caspase-1 in NASH development. Knockout mice (caspases 1/11 and Nlrp3) were evaluated with a standard fat diet or a high-fat diet. Caspases 1/11 knockout mice were predisposed to hepatic steatosis irrespective of the type of diet received. The lack of caspases 1/11 was also linked to higher hepatic triacylglycerol levels. Additionally, increased levels of *Proteobacteria* and a higher Firmicutes/Bacteroidetes ratio were found in the gut of caspases 1/11 knockout mice nourished with a high-fat diet [60]. To evaluate the implication of Nlrp3 in the development of NAFLD, Nlrp3 knockout mice were fed with a Western-lifestyle diet with fructose in drinking water or a chow diet. Knock-out Nlrp3 mice showed increased peroxisome proliferator-activated receptor-γ2 expression and triglyceride contents, greater adipose tissue inflammation and histological liver damage, as well as dysbiotic microbiota with bacterial translocation, related to an increase in TLR4 and TLR9 hepatic expression. After antibiotic treatment, the abundance of Gram-negative species and translocation of bacteria were reduced, and adverse effects were repaired both in the liver and adipose tissue [61]. Higher TLR4, TLR9, MyD88, Casp1, and NLPR3 expression levels were also found in animals that received a high-fat and choline-deficient diet. Concerning the composition of gut microbiota, the number of operational taxonomic units and the Bray-Curtis dissimilarity index were significantly different compared with the control group [62].

A recent study [63] reported that bile acids modulated through microbial modulation were critical to normalize obesity-induced metabolic disorders in hamsters. In addition, these authors found that the elimination of the gut microbiota increased hepatic bile acid synthesis and inhibited the microbial dihydroxylation and deconjugation in the gut [63]. Mice fed with either a low-fat diet with casein or a high fat-diet with casein, fish, or mutton protein were analyzed. The different types of protein in the high-fat diet significantly impacted the gut microbiota composition, with changes in *Prevotellaceae* UCG-003, *Ruminococcaceae* UCG-005, *Desulfovibrio*, the *Lachnospiraceae* NK4A136 group, *Lactobacillus*, and *Akkermansia*, intestinal inflammatory gene expression, serum endotoxin level, and changes in nine metabolites associated with hepatic MetS [64]. The hepatocyte-specific loss of the bacterial wall sensor nucleotide-binding oligomerization domain-containing (NOD) 2 transformed the gut microbiota composition, augmenting *Clostridiales* and diminishing *Erysipelotrichaceae*, among other taxa, verifying that NOD2 protects diet-induced NAFLD in mice [65]. Rats fed a high-fat and choline-deficient diet (a well-established nutritional model of NASH) showed a significantly higher delta Lee index, abdominal adipose tissue, and abdominal circumference, as well as other biochemical parameters compared to control standard diet animals. In this line, the methionine–choline-deficient diet induced steatohepatitis and a decrease in the gut microbiota diversity although these changes in gut microbiota differ from those observed in human subjects with NASH [66]. Using a different nutritional model, a high-fat high-cholesterol diet stimulated fatty liver, steatohepatitis, fibrosis, and NAFLD–HCC development, while a high-fat low-cholesterol diet induced only hepatic steatosis in mice. Microbiota composition was also altered in this model, showing an increase in *Mucispirillum*, *Desulfovibrio*, *Anaerotruncus*, and *Desulfovibrionaceae* and low levels of *Bifidobacterium* and *Bacteroides* in the high-fat high-cholesterol diet-fed group. In addition, dietary cholesterol induced changes in gut bacterial metabolites with augmented levels of taurocholic acid and decreased 3-indole propionic acid [67].

The recognized translational model for NAFLD/NASH, i.e., Leiden mice [68,69], was used by Gart et al. to test several energy-dense diets. Animals were fed with chow, butterfat–fructose, lard fat–sucrose, or a diet with lard fat–sucrose and fructose water. General variations in microbiota were detected in all groups as well as modifications in plasma short-chain fatty acids (SCFAs) [70].

On the other hand, proton pump inhibitors have been shown to stimulate the progression of alcoholic liver disease, NAFLD, and NASH in mice by growing *Enterococcus* spp. and *Enterococcus faecalis* [71].

Germ-free mice received microbiota from four donors subjected to different diets, i.e., (1) control diet, (2) high-fat diet-responders, (3) high-fat diet non-responders, and (4) a quercetin-supplemented high-fat diet to investigate changes in NAFLD development. The high-fat diet non-responders and quercetin-supplemented high-fat diet groups had higher levels of *Desulfovibrio* and *Oscillospira*, diminished levels of *Bacteroides* and *Oribacterium*, higher stimulation of hepatic bile acid transporters, and suppression of hepatic lipogenic and bile acid synthesis genes. The authors suggested a hepatoprotective effect in the high-fat diet non-responders and of the quercetin-supplemented high-fat diet [72].

Initial infant gut colonization through microbes plays an important role in immunity and metabolic function [73]. Germ-free mice were colonized with stool microbes from 2-week-old human infants born to obese or normal-weight mothers. The stool from infants had higher hepatic gene expression for endoplasmic reticulum stress and innate immunity together with periportal inflammation histological signs, similar to pediatric cases of NAFLD. These results postulate useful data supporting the role of maternal obesity-associated infant dysbiosis in children with obesity and NAFLD [73].

Bearing in mind all the aforementioned, studies in animals seem to be a good tool to explore the implication of microbiota in NAFLD/NASH even when many limitations to extrapolating information to humans have to be considered. Overall, animal models are very helpful to shed light on the effect of alterations in the gut microbiota in hepatic disease, especially when NAFLD patients present different severities, heterogeneous lesions, and variable demographic characteristics (e.g., age, sex, or ethnicity). Additionally, the complexity of human behavior, diet, physical activity, environment, psychological stress, or genetics affects the gut microbiota, which may lead to discrepancies and controversial results.

Although animal models of NAFLD/NASH do not always show all the histological alterations compared with humans (e.g., hepatocyte ballooning) and the microbiota are not strictly the same between species, the effect of an unbalanced microbiota on hepatic disease or the possibly of providing proof of concept may be more reasonable through appraisal of experimental models. Moreover, animal studies have advantages such as reduced biological variation or easy housing and monitoring or control of the diet/environment—all factors with a great impact on the microbiota profile.

### 4.2. Human Studies (Adults and Children)

It has been described that the relationship between *Bacteroides* and a Western-type diet has a pro-inflammatory effect related to the pathogenesis of NASH [44]. In addition, it is known that patients with NAFLD show a different bacterial community with lower biodiversity compared with healthy individuals [74]. Non-virulent endotoxin-producing strains of pathogenic species overgrowing in the gut of patients with obesity can act as contributory agents for the initiation of NAFLD. The most important molecular mechanism is mediated through the TLR4 receptor; this receptor can modulate the different steps in NAFLD evolution and related metabolic disorders [75]. Concerning the NASHmicrobiota association, *Bacteroides* abundance was significantly increased in NASH patients, whereas *Prevotella* was reduced. *Ruminococcus* was higher in patients with fibrosis, and the *Bacteroides* relative abundance was independently related to NASH. Alterations in metabolic pathways were associated with carbohydrate, lipid, and amino acid metabolism [76]. 

Metagenomic analyses of diagnosed patients with NAFLD, NASH, and obesity compared with control individuals revealed an increase in Actinobacteria and reduced Bacteroidetes in NAFLD patients. In NASH patients, low *Oscillospira* levels were related to high *Dorea* and *Ruminococcus* and high 2-butanone and 4-methyl-2-pentanone levels [42]. In the same line, another study reported decreased Bacteroidetes and *Ruminococcaceae* as well as increased *Lactobacillaceae* and *Veillonellaceae* and *Dorea* abundances in NAFLD patients [77]. In addition, in patients with advanced fibrosis, serum and fecal bile acid quantities increased, with serum glycocholic acid fecal deoxycholic acid levels associated with *Bacteroidaceae* and *Lachnospiraceae* [78]. In concordance with these results, *Ruminococcaceae* and *Veillonellaceae* were the central microbiota related to fibrosis severity in non-obese subjects, and bile acids and propionate were elevated in non-obese patients with significant fibrosis [79]. In the same line, a prospective cross-sectional study was conducted to characterize the differences between non-obese adults with and without NAFLD in fecal microbiota. It revealed that NAFLD patients presented additional Bacteroidetes and fewer Firmicutes that produced SCFAs, and 7α-dehydroxylating bacteria decreased. By contrast Gram-negative bacteria were predominant in NAFLD patients [80].

In children with NAFLD, alanine aminotransferase activity and concentrations of some inflammation and insulin resistance markers were significantly higher in plasma as well as other variables related to the bacterial response. By contrast, soluble CD14 serum, D-lactate plasma levels, and small intestinal bacterial overgrowth did not vary [81]. In a study performed in one hundred and twenty-five children and adolescents who were overweight and obese aged 10–18 years and 120 children and adolescents matched for age, those with obesity and who were overweight presented intestinal dysbiosis (37.6%), and 62.4% were small intestinal bacterial overgrowth negative. Children with combined obesity or who were overweight and with a positive small intestinal bacterial overgrowth showed indications of impaired liver function (i.e., high levels of aminotransferases, aspartate aminotransferases, alanine aminotransferase) as well as hypertension and MetS [82]. Low alpha diversity has also been related to a high hepatic fat fraction in adolescents. The most important taxa associated with these changes were *Bilophila* and *Paraprevotella* [83].Table 1 summarizes the principal information from gut microbiota and non-alcoholic steatohepatitis studies.

**Table 1:** Main information from gut microbiota and non-alcoholic steatohepatitis studies.

Reference	Animal Model/Study Population	Main Changes/Microbiota Alterations
Chen et al., 2019 [58]	Knockout of SIRT3 HFD in mice	Impairment of dysbiosis after a HFD with ↑*Desulfovibrio*, *Oscillibacter* and ↓*Alloprevotella* abundances, ↑ LPS levels and dysfunction, ↑cannabinoid receptor 1 and 2 expressions in the colon and liver
Rahman et al., 2016 [47]	Knockout of the F11 receptor gene in mice	Developed NASH features, ↑levels of inflammatory microbial taxa related to Firmicutes and *Proteobacteria*
de Sant’Ana et al., 2019 [60]	Knockout mice (caspases 1/11 and Nlrp3 HFD)	↑*Proteobacteria* and Firmicutes/Bacteroidetes ratio were found in the gut of caspases 1/11 knock-out mice
Pierantonelli et al., 2017 [61]	Nlrp3 knock-out mice	After antibiotic treatment, the abundance of Gram-negative species and translocation of bacteria were reduced, and adverse effects were repaired both in the liver and adipose tissue
Sun et al., 2019 [63]	Hamsters	Microbial modulation of bile acids was modulated, which was key in ameliorating obesity-induced metabolic disorders
Ahmad et al., 2020 [64]	Mice C57BL/6J HFD	HFD induced changes in *Prevotellaceae* UCG-003, *Ruminococcaceae* UCG-005, *Desulfovibrio*, the *Lachnospiraceae* NK4A136 group, *Lactobacillus*, and *Akkermansia*
Cavallari et al., 2020 [65]	Mice (NOD2 knockout)	↑*Clostridiales* and ↓*Erysipelotrichaceae,* NOD2 protection of NAFLD features
Schneider et al., 2019 [66]	Rats with NASH induced by a methionine–choline deficient diet	↓gut microbiota diversity (different from that observed in human NASH subjects)
Zhang et al., 2020 [67]	Mice, C57BL/6 male, high-fat high-cholesterol diet	*↑Mucispirillum*, *Desulfovibrio*, *Anaerotruncus*, and *Desulfovibrionaceae* and ↓*Bifidobacterium* and *Bacteroides*
Gart et al., 2018 [70]	Leiden mice	General variations in microbiota modifications in plasma and short-chain fatty acids
Llorente et al., 2017 [71]	Sublytic *Atp4a^Sl/Sl^* mice, Proton pump inhibitors	↑progression of liver disease, ↑ *Enterococcus* spp., ↑*Enterococcus faecalis*
Petrov et al., 2019 [72]	Germ-free mice, High-fat diet non-responder, Quercetin-supplemented HFD	↑*Desulfovibrio* and *Oscillospira*, ↓*Bacteroides* and *Oribacterium*, ↑stimulation of hepatic bile acid transporters ↓hepatic lipogenic and bile acid synthesis genes
Boursier et al., 2016 [76]	Humans, NASH patients, NASH patients+ fibrosis	↑*Bacteroides* abundance ↓*Prevotella,*↑*Ruminococcus*
Del Chierico et al., 2017 [42]	Humans with NASH	↓*Oscillospira* levels related to ↑*Dorea* and *Ruminococcus* and high 2-butanone and 4-methyl-2-pentanone levels
Adams et al., 2020 [78]	Human patients with advanced fibrosis	↑serum and fecal bile acid quantities Serum glycocholic acid fecal deoxycholic acid levels were associated with *Bacteroidaceae* and *Lachnospiraceae*
Lee et al., 2020 [79]	Fibrosis in non-obese human subjects	Fibrosis severity associated with *Ruminococcaceae* and *Veillonellaceae,* ↑bile acids and propionate were elevated in non-obese patients with significant fibrosis
Belei et al., 2017 [82]	Children with combined obesity or who were overweight and had positive small intestinal bacterial overgrowth	Impaired liver function, Hypertension, Metabolic syndrome
Stanislawski et al., 2018 [83]	Adolescents	↓alpha diversity related to a high hepatic fat fraction in adolescents. Altered *Bilophila* and *Paraprevotella* levels

Abbreviations: HFD, high-fat diet; LPS, lipopolysaccharide; NAFLD, non-alcoholic fatty liver disease; NASH, non-alcoholic steatohepatitis; Nlrp3, NLR family pyrin domain containing 3; NOD2, nucleotide-binding oligomerization domain containing protein 2; SIRT3, NAD-dependent deacetylase sirtuin-3; ↑means increment; ↓means reduction.

## 5. Strategies to Treat Non-Alcoholic Steatohepatitis and the Implication for the Microbiota

NASH is one of the most recurrent causes of cirrhosis. Since the number of approved drugs and their effectivity are limited, it is fundamental to look for beneficial methods that can lead to the prevention or reversal of the progression of NASH [84,85]. In addition, the modulation of gut microbiota brings added benefits by itself or in combination with other interventions (i.e., diet, exercise, bariatric surgery, probiotics administration, etc.). This is a field of growing interest for the scientific community widely approached in the last years by the authors. In the present section, we remark on some studies and results in this regard.

### 5.1. Bariatric Surgery

Bariatric surgery is effective in obesity care and causes prolonged weight loss with potential decreases in hepatic fat, inflammation, and fibrosis [86,87]. A systematic review and meta-analysis [88] in 2008 considered the effects on histology after bariatric surgery in NAFLD patients. The authors concluded that fibrosis, steatohepatitis, and steatosis features were enhanced in most patients succeeding weight loss after bariatric surgery. Most studies described favorable effects on steatosis, and more than half of the studies revealed significant enhancements in histological inflammation. Recently, a systematic review and meta-analysis included forty-three studies divided into behavioral weight loss programs, pharmacotherapy, and bariatric surgery studies with 2809 participants. The authors concluded there is a dose-response association with liver inflammation, ballooning, and NAFLD or NASH resolution; however, there were incomplete data of a dose-response association with NAFLD activity score or fibrosis [89]. 

Evidence shows that the gut microbiota is a key mediator of the metabolic beneficial effects and weight loss observed after bariatric surgery. In this line, significant changes in the microbiota and related genes have been reported after this procedure [90,91,92]. For instance, a study performed on obese patients reported increased gut microbial diversity and alterations in the relative abundances of 31 species after being submitted to bariatric surgery [93]. Suggested mechanisms for the reported changes in microbiota are (1) food choices and preferences, reduction of food consumption, and nutrient malabsorption or diet therapy after surgery; (2) Changes in bile acids that alter the 7α-dehydroxylation capacity of the intestinal microbiota and impair the synthesis of the secondary bile acids; (3) changes in hormones related to both energy metabolism and microbiota such as leptin and ghrelin; (4) pH changes along the stomach induced by bariatric surgery. In this sense, a high pH can strongly affect microbiota (e.g., Bacteroidetes decrease due to pH changes after bariatric surgery, while Firmicutes and Actinobacteria increase) [94].

### 5.2. Physical Exercise as a Potential Treatment for NAFLD/NASH

Given the lack of pharmacologically approved treatment, physical activity and exercise is one of the most promising modifiable lifestyle components related to NAFLD/NASH development [95,96,97]. Indeed, physical inactivity is related to the progression of the disease [98,99], being one of the main problems for these subjects. Previous studies suggest that physical exercise benefits the prevention of several metabolic risk factors related to NAFLD, such as insulin resistance, dyslipidemia, and hypertension, reducing intrahepatic lipids [100]. In this line, the most extensive and simple outcome related to the benefits of exercise is weight loss, with the potential to attenuate or reverse the course of disease [100,101]. Despite this, physical exercise provides benefits to the management of NALFD even in the absence of weight loss, suggesting other mechanisms by which the exercise confers benefits against NAFLD/NASH [100,102]. In terms of metabolism, physical exercise generates a proliferation of angiogenic factors and proliferation of endothelial cells which induce an increase in capillarization, which favors the consumption of fatty acids, reducing their entry into the liver [103]. This type of disease is commonly associated with obese and diabetic people; therefore, it is of vital importance to lose weight, given that there is evidence that excess liver fat (independent of NASH) is associated with increased cardiometabolic risk [104]. Although there is no precise training program, it is known that physical exercise is capable of modulating hepatic steatosis, improving insulin sensitivity or affecting body composition of patients with NAFLD/NASH [105], even without dietary intervention [100].

#### Microbiota Role in the Relationship of Physical Exercise with NAFLD/NASH

Physical exercise has been demonstrated to impact the composition and functional capacity of microbiota with potential health benefits [106,107,108,109]. Thus, it appears that the benefits of physical exercise could have a common link through the intestinal microbiota. The taxonomic changes in the microbiome through physical exercise have been analyzed in depth [106]. Although the field remains to be further investigated, a recent study pointed out these changes in the intestinal microbiota caused by exercise could be beneficial for NAFLD patients [96]. Thus, the principal evidence is provided by studies on obesity or diabetic patients, suggesting a close relationship with NAFLD/NASH patients. Indeed, it was reported that such alterations in the intestinal microbiota were associated with improved circulating insulin, LDL cholesterol, liver mass, and liver triglycerides [110]. In rodent models, a high-fat diet induced gut microbiota dysbiosis and reduced the relative abundance of *Parabacteroides, Flavobacterium*, and *Alkaliphilus* [96]. Moreover, the practice of physical exercise corrected the imbalanced microbiota composition, reaching similar values to the control, which could be associated with a protective effect against early obesity and NAFLD [96]. In addition, physical exercise programs did show increased relative abundances of *Verrucomicrobia* and decreased *Proteobacteria* in overweight women [111], as well as a decrease in the Firmicutes/Bacteroidetes ratio in T2DM patients [112].

In addition, the concomitant effect of exercise on the gut in NAFLD/NASH patients would be related to the close relationship between the gut and liver. The microbiome and metabolites of the gut can directly reach the liver, being modulated by the gut barrier permeability, which is profoundly affected in these patients, as we discussed in our previous study [28]. The effects of physical exercise on endotoxemia and metabolites related to the gut are also potential mechanisms related to improvements in NAFLD/NASH [112,113]. For example, physical exercise increased the abundance of SCFAs, butyrate, and other SCFAs [109]. Furthermore, exercise exerts an effect on other gut-derived metabolites related to hepatic metabolism such as bile acid, choline, and ethanol [113]. On the other hand, exercise restores these genetic capacities to the level of control mice, possibly contributing to improved metabolic alterations, including NAFLD [114]. This improvement is mainly related to a decrease in the expression of genes involved in lipid metabolism, such as SREBP-1c, FAT/Cd36, and C/EBPa [96]. Interestingly, a recent study compared the effect of exercise vs. caloric restriction in obesity-prone HFD-fed rats, showing that only physical exercise increased insulin sensitivity and achieved greater LDL reduction, mainly due to exercise-induced microbiome modifications [110]. Indeed, these changes could be due to a reduced abundance of the *Bacteroidales S24-7* and *Rikenellaceae* families, which positively correlated with liver triglycerides [110]. However, the direct influence of physical exercise on changes in the microbiome has not been studied extensively in NAFLD/NASH patients. A recent study extensively discussed the effect of physical exercise on the microbiome in NAFLD/NASH [113]. Nonetheless it has not been defined what type of training generates a greater effect, possibly due to the fact that moderately intense physical activity may quickly lead to a reduction in intrahepatic lipid contents [115].

In summary, physical exercise has been shown to improve hepatic steatosis in NAFLD and should be included as part of the clinical care of all patients, with several metabolic mechanisms proposed. In addition, despite physical exercise being one of the proposed mechanisms, the effect of the specific role of exercise on microbiota in NAFLD/NASH must be clarified. However, it seems that total exercise duration and amount might be important for ameliorating liver steatosis [116]. Thus, the principal recommendation is achieving the recent physical activity recommendation, which consists of 150–300 min of moderate–intensive physical activity per week, with the inclusion of both aerobic and muscle-strengthening activities. In addition, individuals should start with small amounts of physical activity, and some physical activity is better than none, especially for those not currently meeting these recommendations; further, reducing sedentary behaviors are recommend [113,117,118].

### 5.3. Diet Calorie-Restricted and the Mediterranean Diet

Modification of host-microbiota interactions with personalized nutrition is a new therapeutic opportunity for both disease control and prevention. The function and composition of the intestinal microbiota are formed from infancy when the individual is colonized by bacteria from the parents and the immediate environment, a route that strongly impacts the microbiota composition in adulthood [119,120]. On the other hand, it is well known that high-fat, high-sugar, hypercaloric diets raise the hepatic steatosis risk [121]. Weight loss accomplished through caloric restriction decreases hepatic inflammation and fibrosis and reduces NASH [122]. Studies have shown that a weight loss of 7–10% ends in improvements in the nonalcoholic fatty liver disease activity score, and a weight loss of  ≥ 10% produces a resolution of 90% for NASH, 45% fibrosis regression, and a 100% steatosis resolution [122]. Diet calorie-restriction is the most essential element in NASH nutritional interventions [123]. The Mediterranean diet was recommended for NAFLD patients by the recent European Association for the Study of the Liver; the European Association for the Study of Diabetes; and the European Association for the Study of Obesity Clinical Practice Guidelines [124] since this diet may contribute to partially restore a healthy gut microbiota [125,126].

### 5.4. Probiotic Supplementation

There is an interaction between the intestinal microbiota, related metabolites, and inflammation factors which control the progression and development of NAFLD. In consequence, modulation of the gut microbiota by probiotics appears to be a safe and sustainable strategy for the treatment of NASH. Probiotic supplementation significantly diminished inflammatory biomarkers such as TNF-α and C-reactive protein in NAFLD patients [127]. A systematic review conducted to evaluate the microbiome targeted in NAFLD patients included twenty-one randomized clinical trials; nine evaluated probiotics, and 12 evaluated synbiotics. Probiotic and synbiotic treatments were related to a significant decrease in alanine aminotransferase activity and liver stiffness measurement through elastography as well as augmented odds of progress in hepatic steatosis [128].

In NASH patients, oligofructose increased *Bifidobacterium*, which was inversely associated with obesity and plasma LPS [129]. The intake of a probiotic cocktail produced changes in the microbiota profile (specifically altering the abundance of pathogenic *Enterobacteria*) and feces’ structure, findings that were associated with the improvement of liver inflammation [130]. Accordingly, *Lactobacillus reuteri* and metronidazole either alone or in combination with metformin were administered to rats with NASH showing beneficial effects on the lipid profile, oxidative stress, liver function, autophagic, and inflammatory biomarkers compared with animals treated only with metformin. In addition, gut microbiota changes were observed in the abundances of Firmicutes and Bacteroidetes and propionate, butyrate, and acetate ratios with the amendment of insulin resistance [131].

### 5.5. Fecal Microbiota Transplantation

Fecal microbiota transplantation (FMT) has been prevalent in recent years. FMT is the transplantation of useful bacteria from feces of healthy donors into the gastrointestinal tract of patients to repair the equilibrium of intestinal microbiota [132,133]. The procedure includes the compilation of filtered stools collected from either a healthy donor or from the recipient himself (autologous FMT) at a specific time before the initiation of disease and associated dysbiosis and its connection to the intestinal tract of a patient suffering from a certain medical condition [134]. FMT is efficient and successful treatment in *Clostridium difficile* infection (CDI) in humans [135,136]. In 2010, the United States Infectious Diseases Society of America and Society for Healthcare Epidemiology of America suggested FMT as a treatment plan for CDI in their clinical guidelines [137]. A recent study in patients with NAFLD has shown that FMT did not improve insulin resistance or hepatic proton density fat fraction, but FMT was able to reduce small intestinal permeability in patients [138]. Besides, there are several studies registered on FMT and liver disease on the clinicaltrials.gov webpage; regarding NASH and FMT, there are five studies with complete registration data, three of them with not recruiting status yet from Spain, the United States and India, and the other two with an unknown status from the United States and India.

## 6. Future Perspectives

Nowadays, NAFLD/NASH has risen to pandemic proportions mainly due to our sedentary lifestyle and Westernized diets with an increasing number of patients developing NASH-related end-stage liver disease. Although NAFLD has become a common disease, its exact cause has not been elucidated, and it is almost certainly not the same in every patient; even the mechanism whereby some patients progress to NASH is still unclear. All this is hampered by the fact that current methods of diagnosis present several limitations and are invasive (e.g., liver biopsy), expensive, inaccurate, or inadequate (e.g., techniques based on image acquisition), or have a low predictive potential (e.g., biochemical markers). In consequence, there is an urgent need for a better understanding of the underlying pathophysiology and diagnosis procedure of NASH. New approaches must consider other NAFLD/NASH-related parameters whose levels and/or alterations would account for the diagnosis, prognostication, and selection of patients for treatment and monitoring.

In recent years, several authors have evaluated whether patients suffering from liver disease present a distinctive microbiota profile. The results extracted from the text undoubtedly highlight differences in the microbiota profile of patients compared with healthy individuals that have been associated with both severity and progression of the hepatic disease. Nevertheless, the current knowledge is still limited and sometimes the sample size is relatively small. To our knowledge, to date, all the authors reported the differences/changes found as “potential biomarkers” and there are no microbiota-related biomarkers currently used in clinical practice. In consequence, more research is needed to validate and establish trustworthy and more precise microbiota-related biomarkers. In our view, results need to show higher reproducibility, eliminating discrepancies in patient cohorts, animal models, comparison groups, or health baseline status.

**Figure 1:**
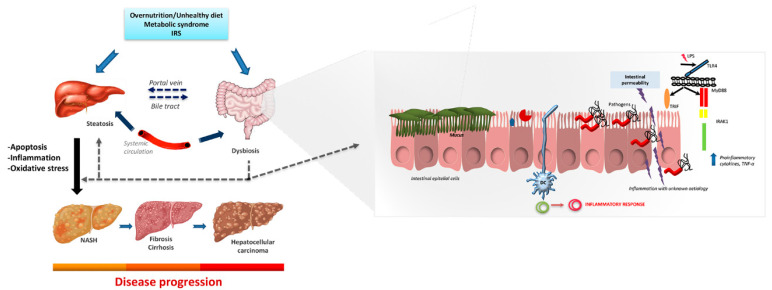
Impact of gut microbiota in hepatic disease. Schematic representation of the main factors involved in the development of NAFLD and the influence of gut microbiota and intestinal permeability through the gut-liver axis. Abbreviations: DC, dendritic cells; IRAK-4; Interleukin 1 Receptor Associated Kinase 4; IRS, insulin resistance syndrome; LPS, lipopolysaccharide; MyD88, Myeloid differentiation primary response 8; NAFLD, Non-alcoholic fatty liver disease; NASH, Nonalcoholic steatohepatitis; TLR4, toll-like receptor; TNF-α, Tumor Necrosis Factor alpha; TRIF, TIR-domain-containing adapter-inducing interferon-β.

Nevertheless, and despite the broad heterogeneity of basic and clinical research on NASH and its systemic complications performed (e.g., bariatric surgery, physical exercise and lifestyle, diet, probiotics or fecal microbiota transplantation), the studies all highlight the crucial role of gut microbiota to maintain the homeostasis of the organism and the implication of the gut–liver axis in the pathogenesis of liver diseases NAFLD, NASH, and HCC and acute liver failure. Moreover, specific microbiota profiles linked to these disorders have been suggested. Microbiota also shift hepatic metabolism through the regulation of hepatic gene expression (Figure 1). Thus, alterations in gut microbiota are emerging as an important tool for determining the occurrence and progression of NAFLD/NASH. Moreover, it is mandatory to take advantage of the current advances in metagenomics techniques. The new knowledge together with proteomics and metabolomics results would be immensely helpful to establish cheaper, novel, and reliable diagnostic biomarkers.

To conclude, the current restrictions in pharmacological treatments for NAFLD/NASH, the limitations of current biomarkers for diagnosis and progression, together with the advancement in the knowledge of NAFLD/NASH microbiota show the need to open a new window the scientific community must explore. New approaches in this sense might provide possible answers in both basic and clinical research to basic questions about prognosis, diagnosis, and monitoring of this huge economic and global health burden.
